# Hidden evolutionary complexity of Nucleo-Cytoplasmic Large DNA viruses of eukaryotes

**DOI:** 10.1186/1743-422X-9-161

**Published:** 2012-08-14

**Authors:** Natalya Yutin, Eugene V Koonin

**Affiliations:** 1 National Center for Biotechnology Information, National Library of Medicine, National Institutes of Health, Bethesda, MD, 20894, USA

## Abstract

**Background:**

The Nucleo-Cytoplasmic Large DNA Viruses (NCLDV) constitute an apparently monophyletic group that consists of at least 6 families of viruses infecting a broad variety of eukaryotic hosts. A comprehensive genome comparison and maximum-likelihood reconstruction of the NCLDV evolution revealed a set of approximately 50 conserved, core genes that could be mapped to the genome of the common ancestor of this class of eukaryotic viruses.

**Results:**

We performed a detailed phylogenetic analysis of these core NCLDV genes and applied the constrained tree approach to show that the majority of the core genes are unlikely to be monophyletic. Several of the core genes have been independently acquired from different sources by different NCLDV lineages whereas for the majority of these genes displacement by homologs from cellular organisms in one or more groups of the NCLDV was demonstrated.

**Conclusions:**

A detailed study of the evolution of the genomic core of the NCLDV reveals substantial complexity and diversity of evolutionary scenarios that was largely unsuspected previously. The phylogenetic coherence between the core genes is sufficient to validate the hypothesis on the evolution of all NCLDV from a common ancestral virus although the set of ancestral genes might be smaller than previously inferred from patterns of gene presence-absence.

## Background

Viruses are ubiquitous, obligate, intracellular parasites of all cellular life forms that rely on the host cell translation system, metabolism and, in many cases, the replication and transcription systems, for their reproduction. There is no evidence that all viruses have a monophyletic origin, at least not under the traditional concept of monophyly. Indeed, not a single gene is conserved in the genomes of all known viruses although a small group of “viral hallmark genes” encoding some of the key proteins involved in genome replication and virion structure formation are shared by large, diverse subsets of viruses [1,2]. However, several large groups of viruses infecting diverse hosts do appear to share common ancestry in the strict sense, that is, to have evolved from the same ancestral virus, as indicated by the conservation of sets of genes encoding proteins responsible for many functions essential for virus reproduction.

One of the largest viral divisions that seem to be monophyletic includes 6 recognized families and a 7^th^ candidate family of viruses with large DNA genomes that infect diverse eukaryotes and are collectively known as Nucleo-Cytoplasmic Large DNA Viruses (NCLDV) [3-6]. The formally recognized NCLDV families are *Poxviridae*, *Asfarviridae*, *Iridoviridae*, *Ascoviridae*, *Phycodnaviridae*, and *Mimiviridae*; in addition, the recently discovered Marseillevirus and the related Lausannevirus could not be assigned to any of the 6 families, and are likely to become founding members of a new family [7,8]. Hereinafter we speak of 7 NCLDV families for the sake of simplicity.

By far the most thoroughly studied group of the NCLDV are the *Poxviridae*, the family of animal viruses that include a major human pathogen, the smallpox virus, important animal pathogens, such as rabbit myxoma virus, as well as vaccinia virus (VACV), one of the best characterized models of molecular biology [9]. Another family of the NCLDV that has recently become a major focus of attention is the *Mimiviridae* that includes giant viruses infecting amoeba and probably algae [10-13]. The genome of the prototype virus of this family, *Acanthamoeba polyphaga* Mimivirus [14], slightly exceeds one megabase (Mb), and other related viruses possess even larger genomes [15,16], so the *Mimiviridae* are undisputed genome size record holders in the virosphere. Indeed, in terms of genome size and complexity, the NCLDV eclipse numerous parasitic bacteria, and approach the simplest free-living prokaryotes.

The NCLDV infect animals and diverse unicellular eukaryotes, and either replicate exclusively in the cytoplasm of the host cells, or encompass both cytoplasmic and nuclear stages in their life cycle. Most of the NCLDV do not strongly depend on the host replication or transcription systems for completing their replication [9,17]. The autonomous life style of the NCLDV is supported by a set of conserved proteins that are encoded in the viral genomes and mediate most of the processes essential for viral reproduction. These essential, conserved proteins include DNA polymerases, helicases, and primases responsible for DNA replication, RNA polymerase subunits and transcription factors that function in transcription initiation and elongation, Holliday junction resolvases and topoisomerases involved in genome DNA processing and maturation, ATPase pumps mediating DNA packaging, molecular chaperones involved in capsid assembly and capsid proteins themselves [3-5]. Although several viral hallmark genes are shared by NCLDV and other large DNA viruses, such as herpesviruses, baculoviruses and some bacteriophages [2], the conservation of the large set of core genes clearly demarcates the NCLDV as a distinct, most likely monophyletic class of viruses [4,6]. More specifically, reconstructions of the ancestral NCLDV genome composition using maximum parsimony and maximum likelihood methods have delineated a set of approximately 50 genes that are inferred to have been responsible for the key functions in the reproduction of the last common ancestor of the NCLDV [5].

The core, presumably ancestral set of the NCLDV genes was delineated using sequence similarity-based methods that have been previously employed for identification of clusters of orthologous genes in diverse cellular life forms. The comparative genomic analysis underlying these reconstructions was deliberately limited to the NCLDV genomes to simplify the analysis and to facilitate detection of distant relationships between viral proteins. Indeed, some of the core NCLDV proteins, such as for example the packaging ATPases and the disulfide chaperones, show only weak sequence similarity between the viral families. Moreover, for some of the NCLDV genes with important functions in virus reproduction, indications of complex evolutionary histories have been obtained. The showcase for such evolutionary complexity is the viral DNA ligase which is represented by two distantly related forms across the NCLDV families. A maximum likelihood reconstruction based on the presence-absence of conserved genes in the viral genomes has implied that one of the two forms, the ATP-dependent DNA ligase, was the ancestral form that was present in the genome of the prototype NCLDV but was replaced by the distantly related NAD-dependent ligase in several viral lineages. However, when the reconstruction was supplemented by phylogenetic analysis of the two forms of DNA ligase, the opposite conclusion has been reached, namely that the NAD –dependent ligase was the ancestral form in NCLDV that was displaced by the ATP-dependent ligase on several independent occasions [18]. This change in perspective occurred because phylogenetic analysis indicated that the ATP-dependent ligases from different lineages of the NCLDV clustered with distinct groups of eukaryotic homologs whereas the NAD–dependent ligases of the NCLDV appeared to be monophyletic. In the same vein, complex phylogenies suggestive of multiple horizontal gene transfer have been observed for several core NCLDV genes such as thymidine kinase or the two subunits of ribonucleotide reductase [19].

Taken together, these findings imply that some of the apparently conserved genes of the NCLDV might actually have complex histories which could include independent (convergent) acquisition of these genes from different cellular organisms as well as displacement of ancestral viral gene by homologs of cellular provenance. This line of reasoning prompted us to perform a comprehensive phylogenetic analysis of the set of the putative ancestral NCLDV genes. Here we present the results of this analysis which suggest that, although the existence of a common ancestor of the NCLDV is beyond reasonable doubt, most of the conserved NCLDV genes indeed had complex evolutionary histories.

## Results

### Approach and rationale

Maximum likelihood reconstruction of the gene repertoire of the putative ancestral NCLDV has revealed a core consisting of approximately 50 viral genes (Table 1). Most of these genes are present in various subsets of the NCLDV genomes rather than all genomes (Additional file 1) but high likelihoods of ancestral provenance have been estimated for each of them, with the absences accordingly attributed to lineage-specific gene loss. We constructed multiple alignments and position-specific scoring matrices for each of the ancestral NCLDV genes and used these to search for homologs from cellular organisms and other viruses. The resulting sequence sets were clustered to include all NCLDV proteins along with representatives of as many major lineages of cellular organisms as possible but also to trim the sets down to a size amenable to detailed phylogenetic analysis (see Methods for the details). For the sequence sets thus selected, ML phylogenetic trees were constructed and examined using the constrained tree approach (see Methods). Specifically, for all trees in which the NCLDV did not appear as a single strongly supported clade, the likelihood of the original tree was compared with the likelihood of a tree constrained for monophyly of NCLDV. When justified, additional constrained trees, with topologies corresponding to plausible evolutionary scenarios, were analyzed for individual genes. Below we present the results of the constrained phylogenetic tree analysis of the inferred ancestral NCLDV genes.

**Table 1:** Evolutionary scenarios for the ancestral NCLDV genes

**NCVOG**[3,4]	**Vaccinia virus gene number**^**a**^	**Functional category**	**# of viral families/ genomes**	**NCVOG annotation**	**Evolutionary scenario from constrained tree analysis**
NCVOG0038	E9L	DNA replication, recombination and repair	6/45	DNA polymerase elongation subunit family B	Monophyletic NCLDV, common origin with baculoviruses and possibly herpesviruses
NCVOG0023	D5R	DNA replication, recombination and repair	6/45	D5-like helicase-primase	Monophyletic NCLDV but displaced by a bacteriophage homolog in phycodnaviruses.
NCVOG1060	G5R	DNA replication, recombination and repair	5/35	FLAP-like endonuclease XPG (cd00128)	Possibly monophyletic NCLDV although displacement by a phage homolog in poxviruses cannot be ruled out; loss in phycodnaviruses and subsequent acquisition of a eukaryotic homolog by *E. huxlei* virus
NCVOG0036	H6R	DNA replication, recombination and repair	3/23	DNA topoisomerase IB	Possibly monophyletic NCLDV
NCVOG0037	None	DNA replication, recombination and repair	6/15	DNA topoisomerase II	Polyphyletic NCLDV, gene acquired from different eukaryotes
NCVOG0035	None	DNA replication, recombination and repair	3/7	NAD + dependent DNA ligase (smart00532)	Monophyletic NCLDV
NCVOG0034	A50R	DNA replication, recombination and repair	4/19	ATP-dependent DNA ligase (pfam01068, PRK01109)	Polyphyletic NCLDV, gene acquired from different eukaryotes
NCVOG0278	A22R	DNA replication, recombination and repair	5/36	RuvC, Holliday junction resolvases (HJRs); cl00243. Extended Pox_A22, Poxvirus A22 family (pfam04848). Marseille virus protein lacks C-term conserved positions.	Insufficient sequence conservation for reliable phylogenetic analysis
NCVOG0004_AP^a^	None	DNA replication, recombination and repair	4/6	AP (apurinic) endonuclease family 2	Strongly supported monophyly of NCLDV
NCVOG0004_NT^a^	None	DNA replication, recombination and repair	3/4	Nucleotidyltransferase (DNA polymerase beta family)	Polyphyletic NCLDV, gene acquired from different eukaryotes
NCVOG1192	None	DNA replication, recombination and repair	4/13	YqaJ viral recombinase family (pfam09588)	Possibly monophyletic NCLDV
NCVOG0024	None	DNA replication, recombination and repair	¾	Superfamily II helicase related to herpesvirus replicative helicase (origin-binding protein UL9), pfam03121	Limited sequence similarity between proteins from different NCLDV; probable polyphyletic origin; possibly not an ancestral NCLDV gene
NCVOG1115	D4R	DNA replication, recombination and repair	3/23	Uracil-DNA glycosylase	Present in only a few NCLDV; uncertain, monophyly of the NCLDV cannot be ruled out
NCVOG0274	J6R	Transcription and RNA processing	6/36	DNA-directed RNA polymerase subunit alpha	Displacement of ancestral NCLDV gene in mimivirus and ASFV with eukaryotic RNAP2 and RNAP1 subunit genes, respectively; loss in most phycodnaviruses. Ancestral NCLDV gene possibly derived from eukaryote RNAP I
NCVOG0271	A24R	Transcription and RNA processing	6/36	DNA-directed RNA polymerase subunit beta	Possibly polyphyletic NCLDV, with one subset derived from RNAP1. and another from RNAP2; likely more recent displacement with RNAP2 in mimivirus; however, monophyly of NCLDV cannot be ruled out; loss in most phycodnaviruses.
NCVOG0273	G5.5R	Transcription and RNA processing	5/15	DNA-directed RNA polymerase subunit 5	Uncertain, not enough sequence conservation for reliable phylogenetic analysis
NCVOG1164	A1L	Transcription and RNA processing	6/44	A1L transcription factor/late transcription factor VLTF-2	Uncertain, not enough sequence conservation for reliable phylogenetic analysis
NCVOG0262	A2L	Transcription and RNA processing	6/45	Poxvirus Late Transcription Factor VLTF3 like	No obvious homologs outside NCLDV (monophyly of NCLDV by default).
NCVOG0261	A7L	Transcription and RNA processing	5/35	Poxvirus early transcription factor (VETF), large subunit (pfam04441)	No obvious homologs outside NCLDV (monophyly of NCLDV by default).
NCVOG0272	E4L	Transcription and RNA processing	6/39	Transcription factor S-II (TFIIS)-domain-containing protein	Uncertain, not enough sequence conservation for reliable phylogenetic analysis
NCVOG1127	One	Transcription and RNA processing	4/11	transcription initiation factor IIB	Strongly supported monophyly of NCLDV
NCVOG0076	A18R	Transcription and RNA processing	6/38	DNA or RNA helicases of superfamily II (COG1061)	Monophyletic NCLDV except for displacement with a eukaryotic homolog in one phycodnavirus and acquisition of a distinct eukaryotic paralog in ASFV
NCVOG0267	I8R	Transcription and RNA processing	3/23	RNA-helicase DExH-NPH-II	Monophyletic NCLDV; independent losses in several NCLDV lineages
NCVOG1117	D1R	Transcription and RNA processing	6/33	mRNA capping enzyme large subunit	Complex, variable domain architecture; apparent monophyly of NCLDV for the conserved methyltransferase domain; guanylyltransferases of apparent distinct eukaryotic origin in a single iridovirus and a single phycodnavirus
NCVOG0236	D9R, D10R	Transcription and RNA processing	6/29	Nudix hydrolase	Uncertain, not enough sequence conservation for reliable phylogenetic analysis
NCVOG1088	None	Transcription and RNA processing	3/13	RNA ligase	Monophyletic NCLDV; common origin with baculovirus and possibly bacteriophage homologs
-	J3R	Transcription and RNA processing	2/16	PolyA polymerase, regulatory subunit	Present only in poxviruses and one mimivirus; however, monophyly strongly supported; possible ancestral gene
NCVOG0276	F4L	Nucleotide metabolism	6/29	Ribonucleotide reductase small subunit	Polyphyletic NCLDV, complex evolutionary scenario; ancestral status uncertain; trees similar for the large and small subunits
NCVOG1353	I4L	Nucleotide metabolism	6/24	ribonucleoside diphosphate reductase, large subunit	Polyphyletic NCLDV, complex evolutionary scenario; ancestral status uncertain; trees similar for the large and small subunits
NCVOG0319	J2R	Nucleotide metabolism	5/20	Thymidine kinase	Most likely polyphyletic NCLDV although monophyly could not be statistically rejected
NCVOG0320	A48R	Nucleotide metabolism	4/21	Thymidylate kinase	Most likely polyphyletic NCLDV although monophyly could not be statistically rejected
NCVOG1068	F2L	Nucleotide metabolism	4/30	dUTPase (cl00493)	Polyphyletic NCLDV, monophyly rejected
NCVOG0022	D13L	Virion structure and morphogenesis	6/45	NCLDV major capsid protein	No homologs outside NCLDV – monophyletic NCLDV by default
NCVOG0249	A32L	Virion structure and morphogenesis	6/45	A32-like packaging ATPase	Only distant homologs outside NCLDV – monophyletic NCLDV by default
NCVOG0211	F9L, L1R	Virion structure and morphogenesis	4/34	myristylated envelope protein	No homologs outside NCLDV – monophyletic NCLDV by default
NCVOG1122	G9R, J5L, A16L	Virion structure and morphogenesis	3/31	Myristylated protein; pfam03003, DUF230	No homologs outside NCLDV – monophyletic NCLDV by default
NCVOG0052	E10R	Virion structure and morphogenesis	6/44	disulfide (thiol) oxidoreductase/isomerase; Erv1 / Alr family (pfam04777)	Monophyletic NCLDV
NCVOG0256	H3L	Virion structure and morphogenesis	2/22	envelope protein, glycosyltransferase	Apparent monophyletic NCLDV but represented only in poxviruses and mimiviruses; independent acquisition cannot be ruled out
NCVOG0040	H1L	Signal transduction, regulation	4/30	cd00127, DSPc, Dual specificity phosphatases (DSP); Ser/Thr and Tyr protein phosphatases	Most likely independent acquisitions by different NCLDV
NCVOG0330	VACWR012, VACWR207	Signal transduction, regulation	5/26	RING-finger-containing E3 ubiquitin ligase (COG5432: RAD18)	Most likely independent acquisition by different groups of the NCLDV; probably not an ancestral gene
NCVOG0329		Signal transduction, regulation	2/3	UBCc, Ubiquitin-conjugating enzyme E2 (cd00195)	Present only in mimivirus and ASFV; independent acquisitions from eukaryotes, NCLDV monophyly rejected; not an ancestral gene
NCVOG0246		Signal transduction, regulation	3/4	Ulp1-like protease/ deubiquitinating enzyme	Uncertain, highly diverged NCLDV sequences
NCVOG0009		Virus-host interactions	3/4	pfam00653: BIR (Baculovirus Inhibitor of apoptosis protein Repeat) domain	Most likely independent acquisition by different groups of the NCLDV; probably not an ancestral gene
NCVOG0012	A33R, A44R, A40R	Virus-host interactions	3/20	C-type lectin: smart00034, cd03594, cd03593, pfam00059, cd00037, pfam05966	Poorly conserved sequence, most likely independent acquisitions by different groups of NCLDV; probably not an ancestral gene
NCVOG1361		Uncharacterized	6/11	T5orf172 domain (pfam10544)	Different domain architectures; most likely independent acquisition by different groups of the NCLDV; probably not an ancestral gene
NCVOG1360	VACWR011, VACWR208	Uncharacterized	3/18	KilA domain (pfam04383); always present at protein N-termini except for mimiviruses. In some proteins fused to the a RING-finger domain.	Different domain architectures; most likely independent acquisition by different groups of the NCLDV; probably not an ancestral gene
NCVOG1424		Uncharacterized	3/6	Uncharacterized domain; found downstream of KilA, BRO, and MSV199 domains. Also found in some baculoviruses.	Different domain architectures; most likely independent acquisition by different groups of the NCLDV; probably not an ancestral gene
NCVOG0010		Uncharacterized	4/11	pfam02498: Bro-N; BRO family, N-terminal domain: This family includes the N-terminus of baculovirus BRO and ALI motif proteins.	Different domain architectures; most likely independent acquisition by different groups of the NCLDV; probably not an ancestral gene
NCVOG0059		Uncharacterized	2/3	FtsJ-like methyltransferase family proteins (pfam01728)	Present only in mimivirus and ASFV; monophyly cannot be ruled out

^a^The systematic gene names are for the Copenhagen strain of Vaccinia virus except for the names starting with VACWR which are from the WR strain because the respective genes are missing/disrupted in the Copenhagen strain.

### Genes involved in genome replication, recombination and repair

**Figure 1:**
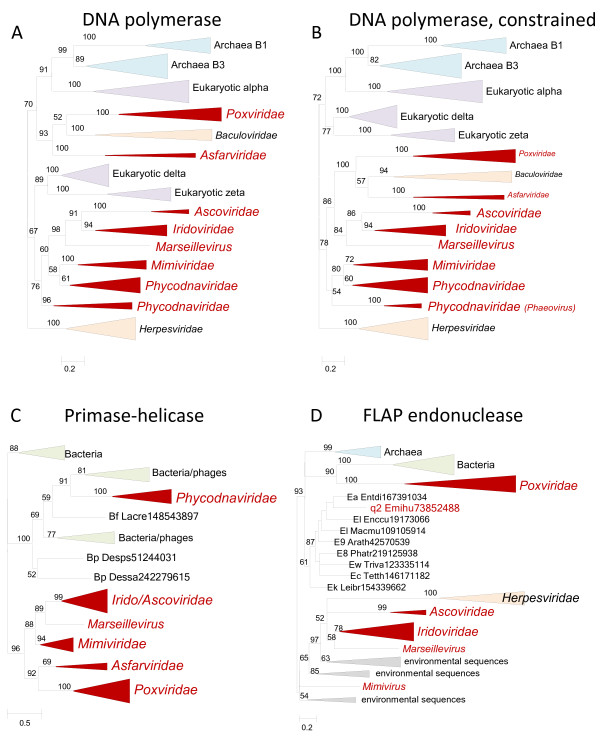
** Phylogenetic trees of ancestral NCLDV genes involved in DNA replication.** (**A**). DNA polymerase, the original ML tree. (**B**). DNA polymerase, constrained tree, monophyly of NCLDV, baculoviruses and herpesviruses enforced. (**C**). Primase-helicase. (**D**). FLAP endonuclease. Branches with bootstrap support less than 50 were collapsed. For each sequence, the species name abbreviation and the gene identification numbers are indicated. Species abbreviations: Arath, *Arabidopsis thaliana*; Desps, *Desulfotalea psychrophila* LSv54; Dessa, *Desulfovibrio salexigens* DSM 2638; Emihu, *Emiliania huxleyi* virus 86; Enccu, *Encephalitozoon cuniculi* GB-M1; Entdi, *Entamoeba dispar* SAW760; Lacre, *Lactobacillus reuteri* DSM 20016; Leibr, *Leishmania braziliensis* MHOM/BR/75/M2904; Macmu, *Macaca mulatta*; Phatr, *Phaeodactylum tricornutum* CCAP 1055/1; Tetth, *Tetrahymena thermophila*; Triva, *Trichomonas vaginalis* G3; Bf, *Firmicutes*; Bp, *Proteobacteria*; E8, *Stramenopiles*; E9, *Viridiplantae*; Ea, *Amoebozoa*; Ec, *Alveolata*; Ek, *Euglenozoa*; El, *Opisthokonta*; Ew, *Parabasalidea*; q2, Coccolithovirus.

The DNA replication of the NCLDV is largely independent of the host replication, and accordingly, genes encoding protein components of the complex replication machinery represent a major part of the NCLDV gene core (Table 1). This is the largest group in the ancestral NCLDV gene set that includes 13 genes two of which, the DNA polymerase (DNAP) and the primase-helicase, are central and indispensable to replication and are shared by all viruses of this class (it has been claimed that the primase-helicase was missing in some phycodnaviruses [20]; however, in the process of NCVOG construction [5], this gene was identified in all complete NCLDV genomes, the high sequence divergence in some of the viruses notwithstanding). The B family of DNAPs, to which the NCLDV polymerases belong, includes the main replicative polymerases of all archaea and eukaryotes as well as many bacterial, archaeal and eukaryotic viruses. The unconstrained phylogenetic tree for the DNAP failed to recover an NCLDV clade. Instead, the majority of the NCLDV DNAPs along with the DNAPs of herpesviruses formed a clade with eukaryotic DNAP delta and zeta; a sister group to this large branch was a clade that consisted of poxvirus, asfarvirus and baculovirus DNAPs (Figure 1A). However, constrained trees in which monophyly of the NCLDV was enforced could not be rejected by statistical tests (Additional file 2). Moreover, among the tested trees, the tree in which the NCLDV clade also included baculoviruses (as the sister group to asfarviruses) and herpesviruses as the sister group to the composite NCLDV-baculovirus clade, had the highest associated likelihood although none of the analyzed tree topologies could be statistically rejected (Figure 1B and Additional file 2). Thus, the results of the constrained tree analysis are compatible with the monophyly of the DNAPs of all NCLDV and moreover of all large DNA viruses of eukaryotes. This conclusion contrasts the results of several previous phylogenetic analyses that failed to recover an NCLDV clade but did not report attempts to analyze constrained trees [21-23]. It seems likely that the apparent non-monophyly of the NCLDV in previous studies was caused by branch length effects, in particular acceleration of evolution in some of the viruses resulting in long branch attraction [24] (note the branch length, in particular for Poxviridae in Figure 1A,B) and possibly other artifacts of phylogenetic analysis. 

Phylogenetic analysis of the second hallmark viral replication gene, the primase-helicase, revealed a strongly supported clade that included 6 of the 7 NCLDV families: phycodnaviruses belonged to a different, also well-supported branch together with diverse bacteriophage and bacterial (most likely, prophage) homologs (Figure 1C). In this case, the constrained tree topology that included an NCLDV clade was rejected at a statistically significant level (Additional file 2). Thus, the result of the phylogenetic analysis of the NCLDV primase-helicase appears to be best compatible with the presence of this gene in the ancestral NCLDV followed by displacement with a homologous and functionally analogous protein from a bacteriophage in the ancestor of phycodnaviruses. This appears to be a typical case of xenologous gene displacement [25]).

The third gene involved in replication that is present in a large majority of the NCLDV encodes a FLAP nuclease, an enzyme that is also ubiquitous in cellular life forms and removes single-stranded overhangs from replication and recombination intermediates. Nucleases of this family are essential for replication and in particular recombination in poxviruses [26] but can also assume other functions such as mRNA degradation as is the case in herpesviruses [27]. The phylogenetic tree of FLAP nucleases did not include an NCLDV clade. Instead, poxviruses clustered with bacterial homologs, the only representative in phycodnaviruses (*Emiliana huxlei* virus) placed within the eukaryotic branch, and the rest of the NCLDV formed a clade with the herpesvirus homologs (Figure 1D). However, a constrained tree with an NCLDV clade (excluding the *Emiliana huxlei* virus) could not be statistically rejected (Additional file 2). The placement of *E. huxlei* virus within the eukaryotic subtree was supported by high boostrap values, and monophyly of this virus with the rest of the NCLDV was weakly supported (AU value <0.1) even if not firmly rejected (Additional file 2). At present we cannot confidently conclude whether the poxvirus FLAP nuclease is monophyletic with those of other NCLDV or evolved through displacement of the ancestral form with a phage homolog. However, even the simplest evolutionary scenario for this gene involves loss of the ancestral gene in phycodnaviruses followed by regain of the eukaryotic homolog by the *E. huxlei* virus.

**Figure 2:**
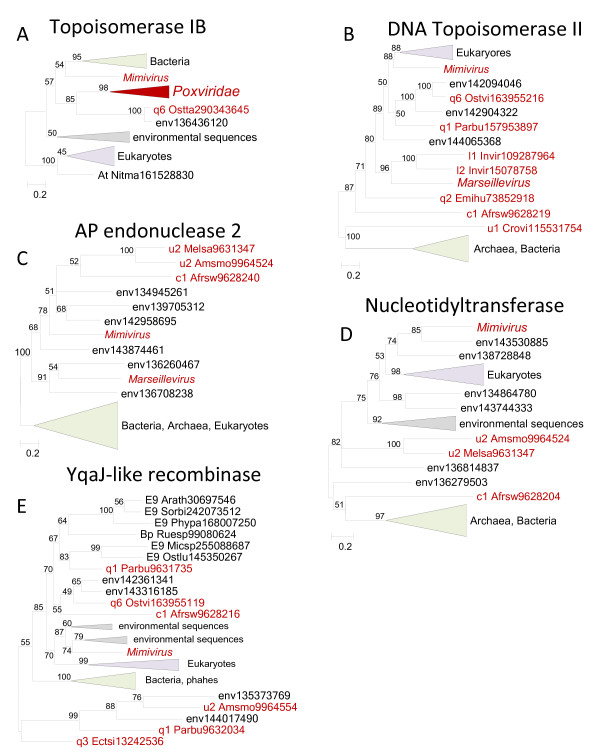
** Phylogenetic trees of ancestral NCLDV genes involved in DNA replication, recombination and repair.** (**A**). Topoisomerase IB. (**B**). DNA Topoisomerase II. (**C**). AP endonuclease 2. (**D**). Nucleotidyltransferase. (**E**). YqaJ-like recombinase. Branches with bootstrap support less than 50 were collapsed. For each sequence, the species name abbreviation and the gene identification numbers are indicated; env stands for sequences retrieved from env_nr database. Species abbreviations: Invir, Invertebrate iridescent virus; Crovi, Crocodilepox virus; Afrsw, African swine fever virus; Amsmo, *Amsacta moorei* entomopoxvirus 'L'; Arath, *Arabidopsis thaliana*; Ectsi, *Ectocarpus siliculosus* virus 1; Emihu, *Emiliania huxleyi* virus 86; Ostlu, *Ostreococcus lucimarinus* CCE9901; Ostta, *Ostreococcus tauri* virus 1; Ostvi, *Ostreococcus* virus OsV5; Parbu, *Paramecium bursaria* Chlorella virus FR483; Phypa, *Physcomitrella patens* subsp. *patens*; Melsa, *Melanoplus sanguinipes* entomopoxvirus; Micsp, *Micromonas* sp. RCC299; Nitma, *Nitrosopumilus maritimus* SCM1; Ruesp, *Ruegeria* sp. TM1040; Sorbi, *Sorghum bicolor*; At, *Thaumarchaeota*; Bp, *Proteobacteria*; E9, *Viridiplantae*; c1, *Asfarviridae*; l1, Chloriridovirus; l2, Iridovirus; q1, Chlorovirus; q2, Coccolithovirus; q3, Phaeovirus; q6, unclassified *Phycodnaviridae*; u1, *Chordopoxvirinae*; u2, *Entomopoxvirinae*.

The remaining genes in this group are less common in NCLDV although the ML reconstruction mapped them to the last common ancestor; the implication of the inferred ancestral status of these genes is that they have been lost on multiple occasions during the evolution of the NCLDV. Topoisomerase IB is represented in poxviruses, one phycodnavirus and mimiviruses. In the phylogenetic tree, poxviruses form a clade with the phycodnavirus but the position of the mimivirus is uncertain (Figure 2A). The monophyly of the NCLDV could not be statistically rejected, so for this gene there is no convincing indication of displacement in any of the NCLDV.

A greater number of NCLDV encode Topoisomerase II that is unrelated to Topoisomerase IB. The phylogenetic tree of Topo II (Figure 2B) did not include an NCLDV clade, and monophyly of the NCLDV could be statistically rejected, with the implication of independent acquisition of the Topo II gene from eukaryotes in mimiviruses and from a prokaryotic source in crocodile poxvirus, with the provenance of this gene in the rest of the NCLDV remaining uncertain (Additional file 2). In principle, the opposite direction of gene transfer, from specific groups of NCLDV to the respective cellular life forms, cannot be ruled out. However, it appears exceptionally unlikely that, for example, a diverse assortment of bacteria and archaea received the Topo II gene specifically from the crocodile poxvirus lineage.

Phylogenetic analysis of apurinic endonuclease 2 (AP2), a repair enzyme represented in a diverse subset of NCLDV, shows unequivocal support for the monophyly of the NCLDV, assuming that the numerous homologous environmental sequences belong to uncharacterized members of the respective NCLDV groups (Figure 2C). In entomopoxviruses, AP2 is fused with a nucleotidyltransferase (a member of the DNA polymerase beta family) whereas ASFV and mimivirus possess a distinct gene encoding a nucleotidyltransferase. However, the phylogenetic tree of nucleotidyltransferases strongly suggests independent acquisitions of this gene by several NCLDV lineages (Figure 2D).

The phylogenetic tree of the YqaJ family recombinase, an enzyme whose role in NCLDV replication and/or repair remains uncertain, is best compatible with multiple acquisitions from bacteriophages although monophyly of the NCLDV could not be ruled out (Figure 2E and Additional file 2).

The unusual case of two distinct DNA ligases was analyzed in detail previously [18]. Surprisingly, the ATP-dependent ligase that is most common among the NCLDV shows clear signs of polyphyletic origin with multiple, independent acquisitions by several virus lineages whereas the less common NAD-dependent ligase appears to be monophyletic, and probably ancestral. A re-analysis performed in the course of this work using an up to date set of sequences provided strong support for this conclusion (Additional file 3).

### Genes involved in transcription and mRNA maturation

**Figure 3:**
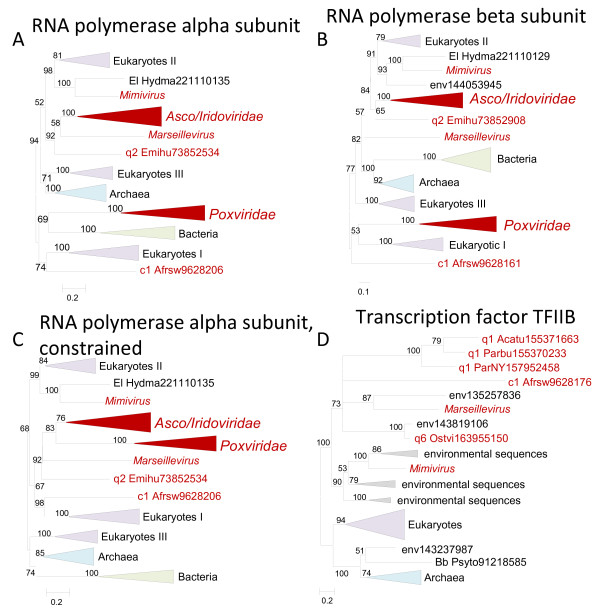
** Phylogenetic trees of ancestral NCLDV genes involved in transcription.** (**A**). RNA polymerase alpha subunit. (**B**). RNA polymerase beta subunit. (**C**). RNA polymerase alpha subunit, constrained tree with an NCLDV clade excluding mimivirus and ASFV. (**D**). Transcription factor TFIIB. Branches with bootstrap support less than 50 were collapsed. For each sequence, the species name abbreviation and the gene identification numbers are indicated; env stands for sequences retrieved from env_nr database. Species abbreviations: Acatu, *Acanthocystis turfacea* Chlorella virus 1; Afrsw, African swine fever virus; Emihu, *Emiliania huxleyi* virus 86; Hydma, *Hydra magnipapillata*; Ostvi, *Ostreococcus* virus OsV5; Parbu, *Paramecium bursaria* Chlorella virus FR483; ParNY, *Paramecium bursaria* Chlorella virus NY2A; Psyto, *Psychroflexus torquis* ATCC 700755; Bb, *Bacteroidetes*; El, *Opisthokonta*; c1, *Asfarviridae*; q1, Chlorovirus; q2, Coccolithovirus; q6, unclassified *Phycodnaviridae.*

All the NCLDV, with the exception of the majority of phycodnaviruses, encode two large subunits of the DNA-dependent RNA polymerase (RNAP) that are also universally conserved in all cellular life forms. It has been reported that in phylogenetic trees the NCLDV RNAP subunits come across as polyphyletic [28,29], and this conclusion is supported by our present analysis (Figure 3A,B). However, for the alpha subunit of the RNAP, the constrained tree in which the NCLDV form a clade, with the exception of the mimivirus and ASFV, actually had the highest likelihood (Figure 3C; Additional file 2). Notably, in this tree the mimivirus gene clustered with the eukaryotic RNAP 2 whereas ASFV clustered with RNAP 1 (Figure 3C), suggestive of two independent displacements of the ancestral NCLDV gene. Similar results were obtained for the RNAP beta subunit: although in this case the original tree with polyphyletic NCLDV had the greatest likelihood, the tree with an NCLDV clade excluding mimivirus and ASFV was nearly as well supported (Additional file 2). As in the case of the alpha subunit, in the RNAP beta subunit tree the mimivirus gene clustered with the eukaryotic RNAP 2 whereas ASFV clustered with RNAP 1 (Figure 3B). 

Three additional RNAP subunits/transcription factors were too divergent to reconstruct reliable phylogenetic trees (Table 1). However, for Transcription factor TFIIB, monophyly of the NCLDV was strongly supported (Figure 3D).

**Figure 4:**
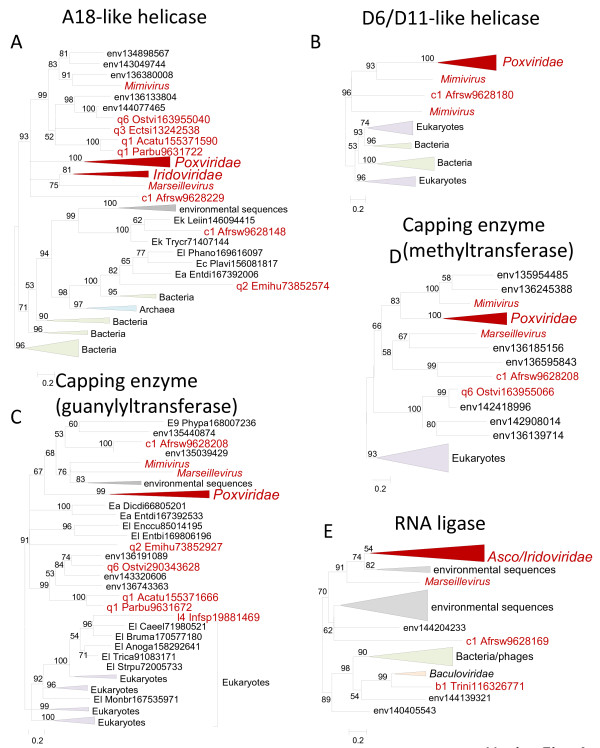
** Phylogenetic trees of ancestral NCLDV genes involved in mRNA processing/maturation.** (**A**). Superfamily 2 helicases homologous to Vaccinia A18. (**B**). Superfamily 2 helicases homologous to Vaccinia D6 and D11. (**C**). Capping enzyme (guanylyltransferase domain). (**D**). Capping enzyme (methyltransferase domain). (**E**). RNA ligase. Branches with bootstrap support less than 50 were collapsed. For each sequence, the species name abbreviation and the gene identification numbers are indicated; env stands for sequences retrieved from env_nr database. Species abbreviations: Phypa, *Physcomitrella patens* subsp. *patens*; Dicdi, *Dictyostelium discoideum* AX4; Entdi, *Entamoeba dispar* SAW760; Plavi, *Plasmodium vivax* SaI-1; Leiin, *Leishmania infantum* JPCM5; Trycr, *Trypanosoma cruzi* strain CL Brener; Anoga, *Anopheles gambiae* str. PEST; Bruma, *Brugia malayi*; Caeel, *Caenorhabditis elegans*; Enccu, *Encephalitozoon cuniculi* GB-M1; Entbi, *Enterocytozoon bieneusi* H348; Monbr, *Monosiga brevicollis* MX1; Phano, *Phaeosphaeria nodorum* SN15; Strpu, *Strongylocentrotus purpuratus*; Trica, *Tribolium castaneum*; Trini, *Trichoplusia ni* ascovirus 2c; Afrsw, African swine fever virus; Infsp, Infectious spleen and kidney necrosis virus; Acatu, *Acanthocystis turfacea* Chlorella virus 1; Parbu, *Paramecium bursaria* Chlorella virus FR483; Emihu, *Emiliania huxleyi* virus 86; Ectsi, *Ectocarpus siliculosus* virus 1; Ostvi, *Ostreococcus* virus OsV5; E9, *Viridiplantae*; Ea, *Amoebozoa*; Ec, *Alveolata*; Ek, *Euglenozoa*; El, *Opisthokonta*; c1, *Asfarviridae*; l4, Megalocytivirus; q1, Chlorovirus; q2, Coccolithovirus; q3, Phaeovirus; q6, unclassified *Phycodnaviridae.*

Phylogenetic analysis of Superfamily 2 helicases homologous to Vaccinia A18 protein (a helicase involved in late transcription [30]) revealed a strongly supported NCLDV clade, with two NCLDV genes placed in other parts of the tree (Figure 4A). These two anomalies include the *E. huxlei* phycodnavirus which fell within a bacteriophage cluster and one of the two members of this helicase family encoded by ASFV. Interestingly, this ASFV protein clustered in the tree and shared domain organization with homologs from Kinetoplastida, suggesting the possibility of gene acquisition from unicellular eukaryotes by an ancestral asfarvirus. Monophyly of this Asfarvirus protein with other NCLDV was strongly rejected (Additional file 2). Thus, two Asfarvirus proteins of this family likely have different origins: one is bona fide NCLDV protein whereas another was acquired from a host. 

Another family of helicases implicated in transcription includes homologs of Vaccinia D6 and D11 which are present in ASFV and in mimivirus. Thus, the presence of an ancestral gene of this family in the NCLDV ancestor implies losses in several groups of viruses. Phylogenetic analysis of this gene strongly supports the monophyly of the NCLDV (Figure 4B).

The capping enzyme of the NCLDV is a complex, three-domain protein. The N-terminal phosphatase domain is too divergent for phylogenetic analysis. Phylogenetic analysis of the other two domains, guanylyltransferase and methyltransferase, identifies an NCLDV clade, to the exclusion of the single representative in iridoviruses that appears to be an independent acquisition of a eukaryotic homolog subsequent to the loss of the ancestral NCLDV gene in iridoviruses (Figure 4C,D).

The RNA ligase is an RNA processing enzyme [31] that is represented in by conserved orthologs iridoviruses, ascoviruses, and Marseillevirus and by a more distant homolog in ASFV; the precise role of this enzyme in the NCLDV reproduction remains unclear. In the phylogenetic tree, the NCLDV ligases of the NCLDV form a well-supported clade (along with many uncharacterized environmental sequences), with the exception of the *Trichoplusia ni* ascovirus which belonged to a clade with baculovirus and diverse bacteriophages (Figure 4E). The constrained tree with an NCLDV clade was confidently statistically rejected (Additional file 2) indicating that in the *Trichoplusia ni* ascovirus the RNA ligase genes was displaced by a homolog from a baculovirus.

Finally, the previously investigated case of the small, regulatory subunit of polyA polymerase is unusual in that this gene is present only in poxviruses which also encode the large, catalytic subunit, and in a single mimivirus strain which lacks the catalytic subunit. Despite this sparse representation, the NCLDV small subunits seem to form a strongly supported clade, suggestive of the possibility that this gene was present in the ancestral NCLDV [15].

### Genes for enzymes of nucleotide metabolism

**Figure 5:**
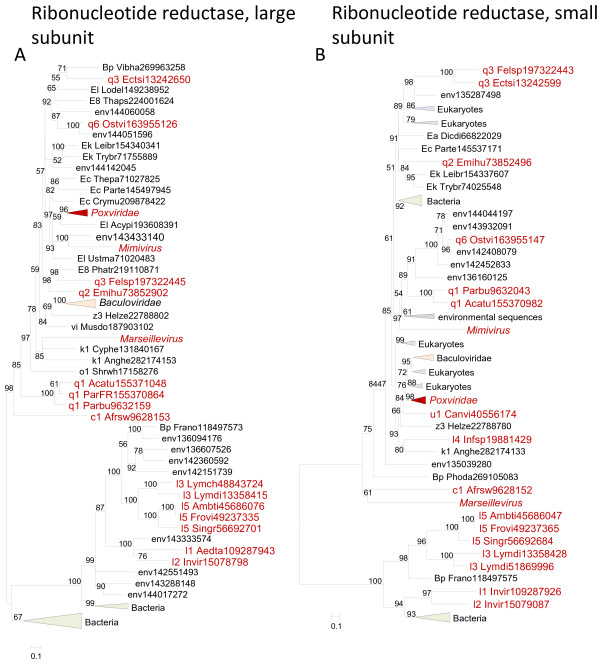
** Phylogenetic trees of the ancestral NCLDV genes for ribonucleotide reductase subunits.** (**A**). Ribonucleotide reductase, large subunit. (**B**). Ribonucleotide reductase, small subunit. Branches with bootstrap support less than 50 were collapsed. For each sequence, the species name abbreviation and the gene identification numbers are indicated; env stands for sequences retrieved from env_nr database. Species abbreviations: Acatu, *Acanthocystis turfacea* Chlorella virus 1; Acypi, *Acyrthosiphon pisum*; Aedta, Invertebrate iridescent virus 3; Afrsw, African swine fever virus; Ambti, *Ambystoma tigrinum* virus; Anghe, *Anguillid* herpesvirus 1; Canvi, Canarypox virus; Crymu, *Cryptosporidium muris* RN66; Cyphe, Cyprinid herpesvirus 3; Dicdi, *Dictyostelium discoideum* AX4; Ectsi, *Ectocarpus siliculosus* virus 1; Emihu, *Emiliania huxleyi* virus 86; Felsp, *Feldmannia* species virus; Frano, *Francisella novicida* U112; Frovi, Frog virus 3; Helze, *Heliothis zea* virus 1; Infsp, Infectious spleen and kidney necrosis virus; l1_Invir, Invertebrate iridescent virus 3; l2_Invir, Invertebrate iridescent virus 6; Leibr, *Leishmania braziliensis* MHOM/BR/75/M2904; Lodel, *Lodderomyces elongisporus* NRRL YB-4239; Lymch, Lymphocystis disease virus - isolate China; Lymdi, Lymphocystis disease virus 1; Musdo, *Musca domestica* salivary gland hypertrophy virus; Ostvi, *Ostreococcus* virus OsV5; Parbu, *Paramecium bursaria* Chlorella virus NY2A; ParFR, *Paramecium bursaria* Chlorella virus FR483; Parte, *Paramecium tetraurelia* strain d4-2; Phatr, *Phaeodactylum tricornutum* CCAP 1055/1; Phoda, *Photobacterium damselae* subsp. *damselae* CIP 102761; Shrwh, Shrimp white spot syndrome virus; Singr, Singapore grouper iridovirus; Thaps, *Thalassiosira pseudonana* CCMP1335; Thepa, *Theileria parva* strain Muguga; Trybr, *Trypanosoma brucei*; Ustma, Ustilago maydis 521; Vibha, *Vibrio harveyi* 1DA3; Bp, *Proteobacteria*; E8, *Stramenopiles*; Ea, *Amoebozoa*; Ec, *Alveolata*; Ek, *Euglenozoa*; El, *Opisthokonta*; c1, *Asfarviridae*; k1, *Herpesvirales*; l1, Chloriridovirus; l2, Iridovirus; l3, Lymphocystivirus; l4, Megalocytivirus; l5, Ranavirus; o1, *Nimaviridae*; q1, Chlorovirus; q2, Coccolithovirus; q3, Phaeovirus; q6, unclassified *Phycodnaviridae*; u1, *Chordopoxvirinae*; vi, Nudivirus; z3, unclassified dsDNA viruses.

Most of the NCLDV encode varying sets of enzymes involved in metabolism of deoxyribonucleotides. These genes are not strictly essential for virus reproduction, given that knockouts are typically viable in cell cultures, but tend to be important *in vivo*. The most common enzyme in this group is ribonucleotide reductase (RR) which consists of a large and a small subunits encoded by two distinct genes. The phylogenetic trees of both the large and the small subunits show complex topologies that are incompatible with monophyly of the NCLDV (Figure 5A,B). Moreover, for both subunits, poxviruses and iridoviruses show clear, distinct affinities, with eukaryotic and bacteriophage homologs, respectively. The provenance of the RR in the other NCLDV is less clear. On the whole, it seems to be a safe conclusion that the RR of the NCLDV are not monophyletic but rather have evolved in a complex evolutionary scenario that involved multiple acquisitions, losses and displacement. Therefore, the results of the ML reconstruction notwithstanding, it is difficult to ascertain whether the last common ancestor of the NCLDV encoded RR.

**Figure 6:**
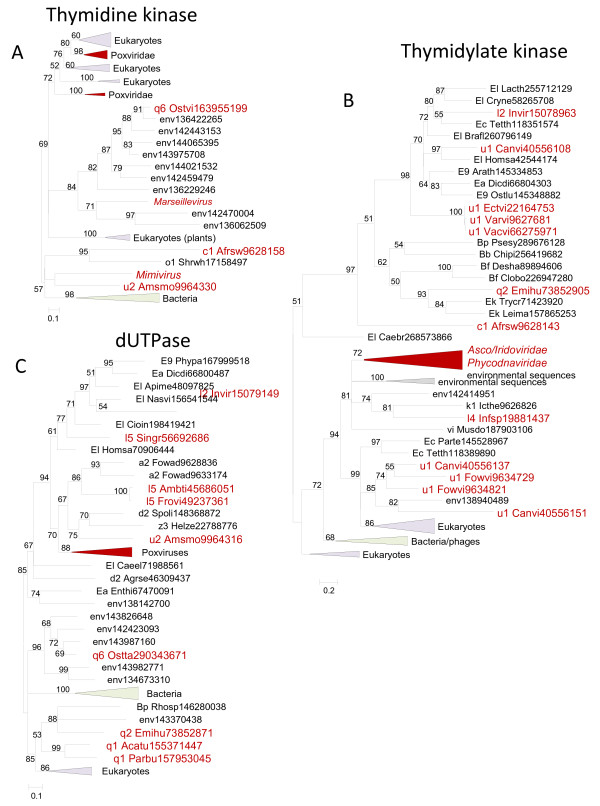
** Phylogenetic trees of ancestral NCLDV genes encoding enzymes of nucleotide metabolism.** (**A**). Thymidine kinase. (**B**). Thymidylate kinase. (**C**). dUTPase. Branches with bootstrap support less than 50 were collapsed. For each sequence, the species name abbreviation and the gene identification numbers are indicated; env stands for sequences retrieved from env_nr database. Species abbreviations: Acatu, *Acanthocystis turfacea* Chlorella virus 1; Afrsw, African swine fever virus; Agrse, *Agrotis segetum* granulovirus; Ambti, *Ambystoma tigrinum* virus; Amsmo, *Amsacta moorei* entomopoxvirus 'L'; Apime, *Apis mellifera*; Arath, *Arabidopsis thaliana*; Brafl, *Branchiostoma floridae*; Caebr, *Caenorhabditis briggsae*; Caeel, *Caenorhabditis elegans*; Canvi, Canarypox virus; Chipi, *Chitinophaga pinensis* DSM 2588; Cioin, *Ciona intestinalis*; Clobo, *Clostridium botulinum* A2 str. Kyoto; Cryne, *Cryptococcus neoformans* var. neoformans JEC21; Desha, *Desulfitobacterium hafniense* Y51; Dicdi, *Dictyostelium discoideum* AX4; Ectvi, *Ectromelia* virus; Emihu, *Emiliania huxleyi* virus 86; Enthi, *Entamoeba histolytica* HM-1:IMSS; Fowad, Fowl adenovirus A; Fowvi, Fowlpox virus; Frovi, Frog virus 3; Helze, *Heliothis zea* virus 1; Homsa, *Homo sapiens*; Icthe, *Ictalurid herpesvirus* 1; Infsp, Infectious spleen and kidney necrosis virus; l2_Invir, Invertebrate iridescent virus 6; Lacth, *Lachancea thermotolerans*; Leima, Leishmania major; Musdo, *Musca domestica* salivary gland hypertrophy virus; Nasvi, *Nasonia vitripennis*; Ostlu, *Ostreococcus lucimarinus* CCE9901; Ostta, *Ostreococcus tauri* virus 1; Ostvi, *Ostreococcus* virus OsV5; Parbu, *Paramecium bursaria* Chlorella virus NY2A; Parte, *Paramecium tetraurelia* strain d4-2; Phypa, *Physcomitrella patens* subsp. *patens*; Psesy, *Pseudomonas syringae* pv. *syringae* FF5; Rhosp, *Rhodobacter sphaeroides* ATCC 17025; Shrwh, Shrimp white spot syndrome virus; Singr, Singapore grouper iridovirus; Spoli, *Spodoptera litura* granulovirus; Tetth, *Tetrahymena thermophila*; Trybr, *Trypanosoma brucei*; Vacvi, Vaccinia virus; Varvi, Variola virus; Bb, *Bacteroidetes*; Bf, *Firmicutes*; Bp, *Proteobacteria*; E9, *Viridiplantae*; Ea, *Amoebozoa*; Ec, *Alveolata*; Ek, *Euglenozoa*; El, *Opisthokonta*; a2, *Adenoviridae*; c1, *Asfarviridae*; d2, *Baculoviridae*; k1, *Herpesvirales*; l2, Iridovirus; l4, Megalocytivirus; l5, Ranavirus; o1, *Nimaviridae*; q1, Chlorovirus; q2, Coccolithovirus; q6, unclassified *Phycodnaviridae*; u1, *Chordopoxvirinae*; u2, *Entomopoxvirinae*; vi, Nudivirus; z3, unclassified dsDNA viruses.

Thymidine kinase (TK) is another major enzyme of dNTP biosynthesis that is present in a large subset of the NCLDV. Phylogenetic analysis reveals three distinct clusters of viral TKs (Figure 6A); a constrained tree containing an NCLDV clade could not be statistically rejected but nevertheless had a lower likelihood than the original tree (Additional file 2). Qualitatively similar results were obtained for Thymidylate kinase (TMPK), the second enzyme of thymidylate biosynthesis (Figure 6B). The dUTPase, an enzyme that functions at the interface of nucleotide metabolism and repair, is present in poxviruses, iridoviruses and many phycodnaviruses, but showed clear signs of polyphyletic origin, including statistically solid rejection of NCLDV monophyly (Figure 6C, Additional file 2). Similar results have been reported from a previous phylogenetic analysis for some of the genes encoding enzymes of nucleotide metabolism [19]. 

**Figure 7:**
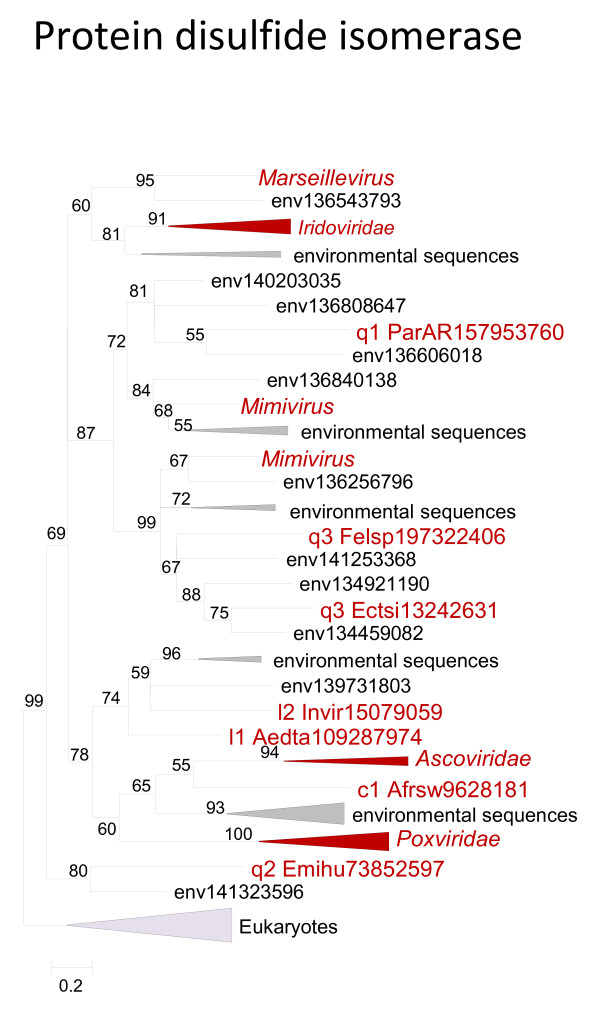
** Phylogenetic tree of an ancestral NCLDV gene encoding an enzyme involved in virion morphogenesis: protein disulfide isomerase.** Branches with bootstrap support less than 50 were collapsed. For each sequence, the species name abbreviation and the gene identification numbers are indicated; env stands for sequences retrieved from env_nr database. Species abbreviations: Afrsw, African swine fever virus; Aedta, Invertebrate iridescent virus 3; Invir, Invertebrate iridescent virus; ParAR, *Paramecium bursaria* Chlorella virus AR158; Emihu, *Emiliania huxleyi* virus 86; Ectsi, *Ectocarpus siliculosus* virus 1; Felsp, *Feldmannia* species virus; c1, Asfarviridae; l1, Chloriridovirus; l2, Iridovirus; q1, Chlorovirus; q2, Coccolithovirus; q3, Phaeovirus.

The majority of the sequences of putative ancestral virion proteins and proteins involved in virus morphogenesis and virus-host interactions were too divergent to construct reliable phylogenetic trees. The only exception was the disulfide isomerase enzyme for which monophyly of the NCLDV (including numerous environmental sequences that presumably belong to uncharacterized viruses) could be demonstrated (Figure 7).

## Discussion

Evolutionary reconstruction using patterns of gene presence-absence in viral genomes has led to the conclusion that the NCLDV represent a monophyletic group of viruses that evolved from a common ancestor which was a virus with genomic complexity comparable to that of the extant NCLDV. Approximately 50 genes that are conserved in different subsets of the NCLDV have been assigned to the ancestral virus genome (Table 1), and it appears likely that the ancestral virus additionally encompassed many lineage-specific genes. In this work we went beyond the phyletic patterns by analyzing phylogenies of the inferred ancestral NCLDV genes along with their homologs from cellular organisms. In almost all cases where the information content of the respective multiple sequence alignments was sufficient for phylogenetic analysis, deviations from the simple pattern of vertical evolution were observed (summarized in Table 1). Strikingly, phylogenetic trees for most of the conserved NCLDV genes failed to show an NCLDV clade although it was not always possible to reject the monophyly of the NCLDV at a statistically significant level.

The results of phylogenetic analysis of viral genes have to be interpreted with caution given the generally fast evolution of viral genomes and the ensuing possibility of artifacts such as long branch attraction. Therefore, we considered the monophyly of the NCLDV to be the most appropriate null hypothesis and generally made conclusions on more complex evolutionary scenarios only when this hypothesis could be rejected using conservative statistical tests of tree topology. Nevertheless, even with this conservative approach, phylogenomic analysis suggests that evolution of the core NCLDV genes, with only a few likely exceptions (DNA polymerase, disulfide isomerase and several other genes; see Table 1), included not only multiple gene losses that were apparent already from the examination of the phyletic patterns, but also multiple cases of xenologous gene displacement [25], i.e. displacement of the ancestral gene by a homologous (and functionally analogous) gene from a different source such as bacteriophage or eukaryote. On some occasions, when a gene is missing in a large group of viruses (such as phycodnaviruses) except for a minority of members of that group, in which it is phylogenetically distinct from homologs in other NCLDV, the sequence of events leading to displacement can be inferred as loss followed by regain. In other cases, such as RR and TK, the course of evolution seems to have been too complex to reconstruct the specific scenario.

Thus, the present analysis reveals layers of hidden complexity in the history of the conserved gene core of the NCLDV that are not apparent from the analysis of patterns of gene presence-absence alone [5]. Although deviations from simple vertical evolution probably occurred in the history of almost all core genes, the results do not invalidate the conclusion on the evolution of all known NCLDV from a single ancestral virus. The high prevalence of gene loss and xenologous gene displacement notwithstanding, for most of the core genes, these events affected the evolution of only a few lineages, consistent with the origin of all NCLDV from a common ancestor, followed by isolated events complicating the evolutionary scenarios. However, for several genes that have been included in the core set on the basis of gene presence-absence patterns, such as the enzymes of DNA precursor metabolism, multiple sources are apparent, so that the ancestral status of these genes becomes uncertain.

Phylogenetic analysis of the core NCDLV genes reveals multiple affinities with genes from eukaryotes, bacteria and bacteriophages. The acquisition of genes from eukaryotic hosts (that might not be the same for ancestral and extant viruses) is not surprising. However, gene transfer to NCLDV, in particular those infecting unicellular eukaryotes, from bacteria and bacteriophages is plausible as well given that diverse parasites and symbionts often coexist within the same eukaryotic host. Indeed, in amoebas, with their large cells and phagocytic life style, such coexistence is the norm, making these organisms veritable ‘melting pots’ of virus evolution [7,32].

The general trend seems to be that bona fide essential genes, are rarely displaced because for these, intermediates lacking the gene are most like non-viable, so if intermediate forms existed, they should have encoded two forms of the respective genes, with the original gene subsequently lost and the xenologous gene retained. This evolutionary scenario might be rare due to the constraints imposed by the requirements for the formation of multisubunit complexes (under the complexity hypothesis of Lake and colleagues [33]), e.g. the replisome. However, the clear-cut xenologous displacement of the primase-helicase gene in phycodnaviruses shows that these obstacles are not insurmountable. In contrast, for genes that are not strictly essential but are beneficial in most virus-host systems, such as the precursor biosynthesis enzymes, parallel loss and regain in multiple lineages seems to be the rule rather than an exception. This pattern can be linked to the viability of evolutionary intermediates lacking the respective genes, at least in the short term.

In addition to cases of xenologous gene displacement, phylogenies of a few core genes point to evolutionary links with other large DNA viruses, such as herpesviruses and baculoviruses, as well as bacteriophages. These observations are compatible with the virus world concept under which viruses are linked through complex networks of evolutionary connections at the level of individual genes and in some cases gene modules, and are also involved in extensive gene exchange with cellular life forms.

## Methods

The NCLDV protein sequences were extracted from the RefSeq database (NCBI, NIH, Bethesda) [34]. The most recently sequenced NCLDV genomes, namely the *Cafeteria roenbergensis* virus [35], the megavirus [16], both of the Mimiviridae family, and Lausannevirus [8], a close relative of Marseille virus, were not included. For each cluster of orthologous NCLDV genes (NCVOG) that has been mapped to the last common ancestor of the NCLDV [5], the following procedure was applied. A representative NCVOG sequence set was constructed by clustering the complete collection of the respective protein sequences using the Blastclust program ( ftp://ftp.ncbi.nih.gov/blast/documents/blastclust.html) and selecting a representative from each cluster of closely related sequences). Two BLASTP runs, one against the Refseq database and the other one against the environmental (env_nr) database at the NCBI, were performed for each representative sequence with the e-value cutoff of 0.1. This liberal cutoff was used in order to incorporate highly diverged homologs. To eliminate potential false positives, all alignments were examined case by case for the conservation of domain architecture and presence of diagnostic motifs. All the sequences from the given NCVOG, the top 20–30 Refseq hits from each domain of the NCBI Taxonomy (Eukaryota, Bacteria, Archaea, and Viruses), and top 10 environmental hits for each query were combined, and nearly identical sequences were eliminated using Blastclust. The resulting sequences were aligned using MUSCLE [36]; gapped columns (more than 30% of gaps) and columns with low information content were removed from the alignment [37]. A preliminary tree was constructed using PhyML [38], with the following parameters: WAG substitution matrix; four relative substitution rate categories; the fraction of invariable sites and the alpha parameter of the gamma distribution of site-specific evolution rates) were automatically selected by PhyML. From this preliminary tree, a subset of sequences representing each clade were selected and re-aligned. In the next step, 8 PhyML runs were performed for each alignment, with 8 substitution models (Blosum62, Dayhoff, JTT, DCMut, RtREV, CpREV, VT, and WAG). The best topology was chosen by the maximum log likelihood among the 8 trees. Bootstrap branch supports (expressed in Expected-Likelihood Weights, ELW) were calculated using TreeFinder [39] for the best PhyML tree using the best matrix. For detailed phylogenetic analysis, whenever appropriate, alternative (constrained) topology ML trees were constructed using TreeFinder [39], with the substitution model found to be the best for a given alignment in the first-round analysis. Tree topologies were compared with TreeFinder using the approximately unbiased (AU) test P value [40].

## Competing interests

The authors declare that they have no competing interests.

## Authors’ contributions

EVK initiated and designed the study; NY collected and analyzed data; EVK wrote the manuscript that was read, edited and approved by both authors.

## Supplementary Material

Additional file 1 The phyletic patterns of absence-presence of the NCLDV core genes in the sequenced NCLDV genomes. Click here for file

Additional file 2 Results of statistical analysis of constrained trees for the core NCLDV genes. Click here for file

Additional file 3 ** Phylogenetic trees for the DNA ligases of the NCLDV.** A. ATP-dependent ligases. B. NAD-dependent ligases. Click here for file
