# Supplementary material for: Hidden evolutionary complexity of Nucleo-Cytoplasmic Large DNA viruses of eukaryotes
Source: Virol J. 2012 Aug 14;9:161. doi: 10.1186/1743-422X-9-161 (PMC3493329; doi:10.1186/1743-422X-9-161)

## Slide 1
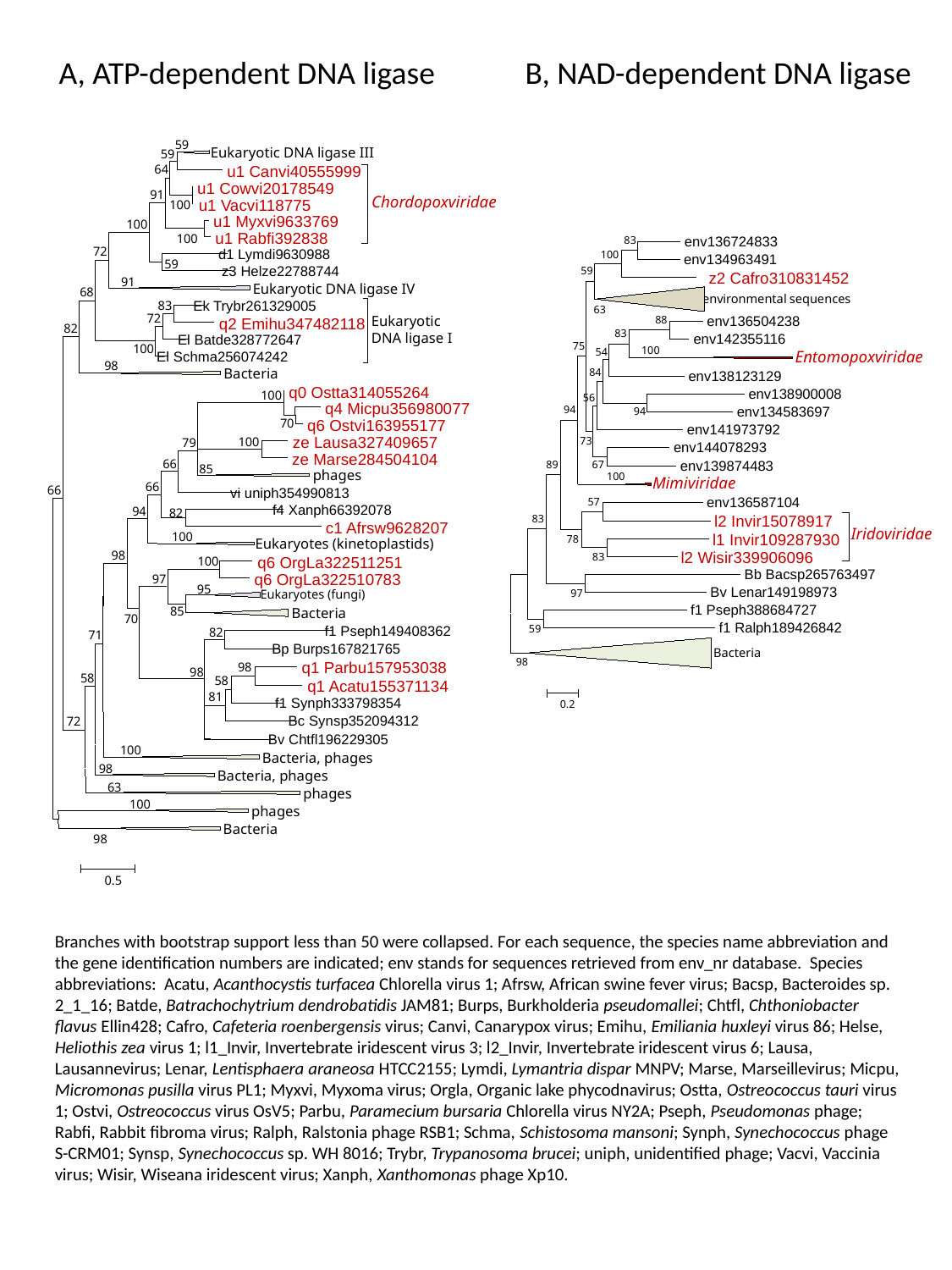

A, ATP-dependent DNA ligase
B, NAD-dependent DNA ligase
59
 Eukaryotic DNA ligase III
59
 u1 Canvi40555999
64
 u1 Cowvi20178549
91
Chordopoxviridae
 u1 Vacvi118775
100
 u1 Myxvi9633769
100
 u1 Rabfi392838
100
72
 d1 Lymdi9630988
59
 z3 Helze22788744
91
 Eukaryotic DNA ligase IV
68
 Ek Trybr261329005
83
72
Eukaryotic
DNA ligase I
 q2 Emihu347482118
82
 El Batde328772647
100
 El Schma256074242
98
 Bacteria
 q0 Ostta314055264
100
 q4 Micpu356980077
 q6 Ostvi163955177
70
 ze Lausa327409657
100
79
 ze Marse284504104
66
85
 phages
66
66
 vi uniph354990813
 f4 Xanph66392078
94
82
 c1 Afrsw9628207
100
 Eukaryotes (kinetoplastids)
98
 q6 OrgLa322511251
100
 q6 OrgLa322510783
97
95
 Eukaryotes (fungi)
85
 Bacteria
71
70
98
58
72
100
 Bacteria, phages
98
 Bacteria, phages
63
 phages
100
 phages
 Bacteria
98
0.5
 f1 Pseph149408362
82
 Bp Burps167821765
 q1 Parbu157953038
98
58
 q1 Acatu155371134
81
 f1 Synph333798354
 Bc Synsp352094312
 Bv Chtfl196229305
 env136724833
83
100
 env134963491
59
 z2 Cafro310831452
 environmental sequences
63
 env136504238
88
83
 env142355116
75
100
54
 Entomopoxviridae
84
 env138123129
 env138900008
56
 env134583697
94
94
 env141973792
73
 env144078293
 env139874483
89
67
100
 Mimiviridae
 env136587104
57
 l2 Invir15078917
83
Iridoviridae
 l1 Invir109287930
78
 l2 Wisir339906096
83
 Bb Bacsp265763497
 Bv Lenar149198973
97
 f1 Pseph388684727
 f1 Ralph189426842
59
 Bacteria
98
0.2
Branches with bootstrap support less than 50 were collapsed. For each sequence, the species name abbreviation and the gene identification numbers are indicated; env stands for sequences retrieved from env_nr database. Species abbreviations: Acatu, Acanthocystis turfacea Chlorella virus 1; Afrsw, African swine fever virus; Bacsp, Bacteroides sp. 2_1_16; Batde, Batrachochytrium dendrobatidis JAM81; Burps, Burkholderia pseudomallei; Chtfl, Chthoniobacter flavus Ellin428; Cafro, Cafeteria roenbergensis virus; Canvi, Canarypox virus; Emihu, Emiliania huxleyi virus 86; Helse, Heliothis zea virus 1; l1_Invir, Invertebrate iridescent virus 3; l2_Invir, Invertebrate iridescent virus 6; Lausa, Lausannevirus; Lenar, Lentisphaera araneosa HTCC2155; Lymdi, Lymantria dispar MNPV; Marse, Marseillevirus; Micpu, Micromonas pusilla virus PL1; Myxvi, Myxoma virus; Orgla, Organic lake phycodnavirus; Ostta, Ostreococcus tauri virus 1; Ostvi, Ostreococcus virus OsV5; Parbu, Paramecium bursaria Chlorella virus NY2A; Pseph, Pseudomonas phage; Rabfi, Rabbit fibroma virus; Ralph, Ralstonia phage RSB1; Schma, Schistosoma mansoni; Synph, Synechococcus phage S-CRM01; Synsp, Synechococcus sp. WH 8016; Trybr, Trypanosoma brucei; uniph, unidentified phage; Vacvi, Vaccinia virus; Wisir, Wiseana iridescent virus; Xanph, Xanthomonas phage Xp10.

## Slide 2
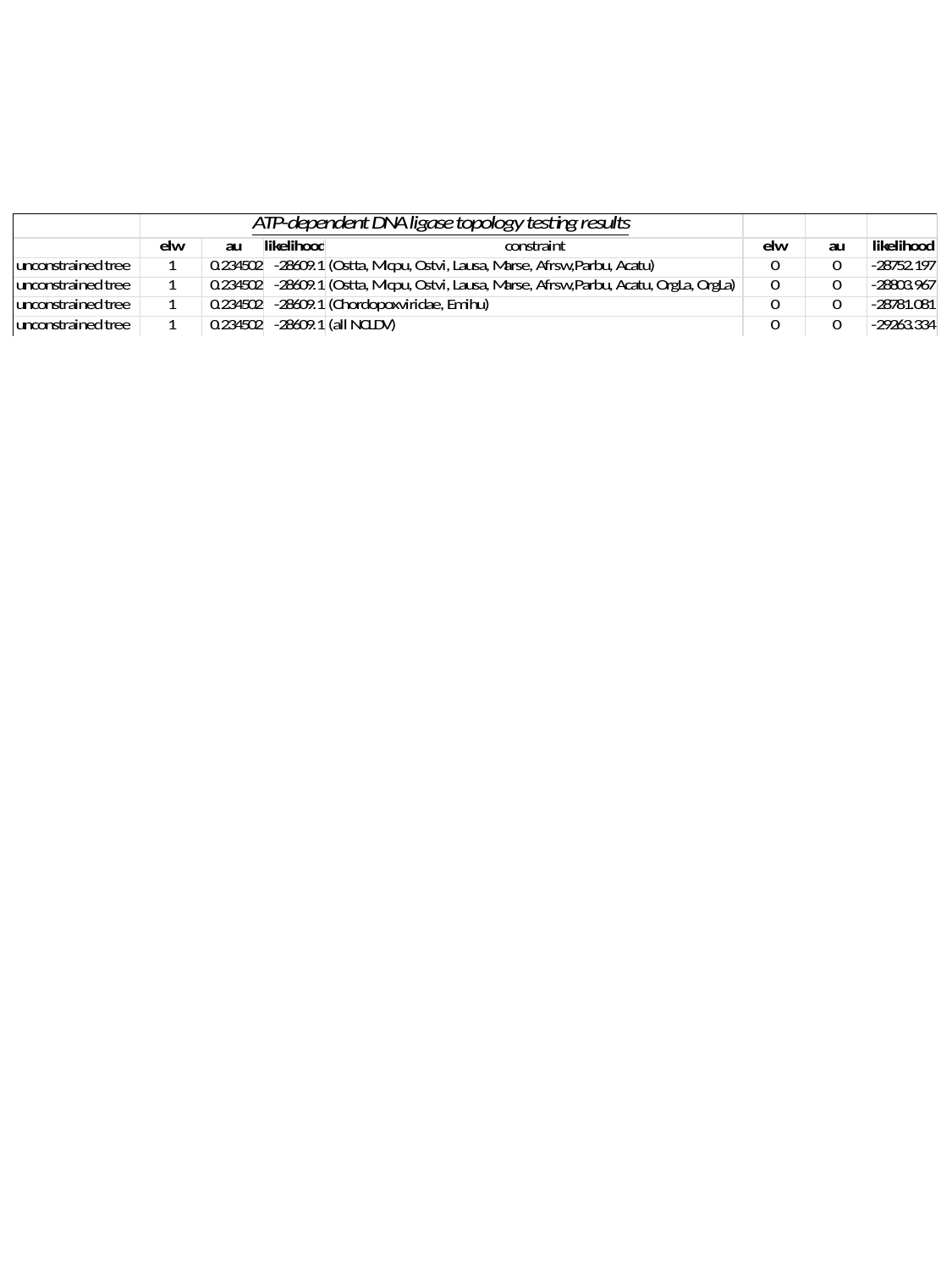

Supplement: Additional file 3 — Phylogenetic trees for the DNA ligases of the NCLDV. A. ATP-dependent ligases. B. NAD-dependent ligases. [file 1743-422X-9-161-S3.ppt]
